# Simulating Sleep Apnea by Exposure to Intermittent Hypoxia Induces Inflammation in the Lung and Liver

**DOI:** 10.1155/2012/879419

**Published:** 2012-11-26

**Authors:** Darlan Pase da Rosa, Luiz Felipe Forgiarini, Diego Baronio, Cristiano Andrade Feijó, Dênis Martinez, Norma Possa Marroni

**Affiliations:** ^1^Ciências Médicas, Programa de Pós-Graduação em Medicina, Universidade Federal do Rio Grande do Sul (UFRGS), 90035-903 Porto Alegre, RS, Brazil; ^2^Hospital de Clínicas de Porto Alegre (HCPA), Universidade Federal do Rio Grande do Sul, 90035-903 Porto Alegre, RS, Brazil; ^3^Faculdade Cenecista de Bento Gonçalves, 95700-973 Bento Gonçalves, RS, Brazil; ^4^Programa de Pós-Graduação em Ciências Pneumológicas, Universidade Federal do Rio Grande do Sul (UFRGS), 90035-903 Porto Alegre, RS, Brazil; ^5^Universidade Luterana do Brasil, 92425-900 Canoas, RS, Brazil

## Abstract

Sleep apnea is a breathing disorder that results from momentary and cyclic collapse of the upper airway, leading to intermittent hypoxia (IH). IH can lead to the formation of free radicals that increase oxidative stress, and this mechanism may explain the association between central sleep apnea and nonalcoholic steatohepatitis. We assessed the level of inflammation in the lung and liver tissue from animals subjected to intermittent hypoxia and simulated sleep apnea. A total of 12 C57BL/6 mice were divided into two groups and then exposed to IH (*n* = 6) or a simulated IH (SIH) (*n* = 6) for 35 days. We observed an increase in oxidative damage and other changes to endogenous antioxidant enzymes in mice exposed to IH. Specifically, the expression of multiple transcription factors, including hypoxia inducible factor (HIF-1*α*), nuclear factor kappa B (NF-*κ*B), and tumor necrosis factor (TNF-*α*), inducible NO synthase (iNOS), vascular endothelial growth factor (VEGF), and cleaved caspase 3 were shown to be increased in the IH group. Overall, we found that exposure to intermittent hypoxia for 35 days by simulating sleep apnea leads to oxidative stress, inflammation, and increased activity of caspase 3 in the liver and lung.

## 1. Introduction

Obstructive sleep apnea (OSA) consists of sleep-disordered breathing. Cyclic episodes result in the momentary closure, partial or complete, of the upper airway at the level of the pharynx. The repeated pauses in breathing can lead to intermittent hypoxia (IH) and increased reactive oxygen species (ROS) [1].

The increase of ROS in OSA is likely due to the repeated oxygen depletion followed by the hyperoxia that develops to restore oxygen pressure (PO_2_). A similar phenomenon is observed in ischemia followed by reperfusion [2–5]. In ischemia/reperfusion, xanthine oxidase generates free radicals in the presence of oxygen, contributing to oxidative stress [6–8].

OSA is associated with chronic liver diseases, such as nonalcoholic steatohepatitis (NASH) [9–16]. Savransky and colleagues demonstrated that IH can act as a “second hit” to liver disease by amplifying the tissue damage induced by a high dose of paracetamol [17, 18]. The injury mechanism, triggered by OSA, appears to be related to the formation of peroxynitrite, depletion of glutathione, and apoptosis of hepatocytes [18].

In OSA, inflammatory factors, such as nuclear factor kappa B (NF-*κ*B), are activated at a systemic level [17, 19]. NF-*κ*B is a master regulator of the inflammatory process, by inhibiting its inhibitor IKK-b, and its activation leads to the increased expression of tumor necrosis factor (TNF-*α*), interleukins 1 and 6, and inducible nitric oxide synthase (iNOS). Alternatively, these factors can be activated by hypoxia inducible factor (HIF-1*α*) [20–23], which results in apoptosis [24]. 

Several studies have shown that OSA is associated with inflammation, NASH, oxidative stress, and apoptosis. This is the first experimental study that evaluated the inflammatory process in the lung and liver with intermittent hypoxia, suggesting that there is a recruitment of inflammatory mediators recognized during ischemia and reperfusion. Here, we investigate the molecular mechanism involved in the lung and liver injury in an animal model of OSA.

## 2. Methods

The experiments were approved and completed according to the Research and Ethics Committee of the Research and Postgraduation at the Hospital de Clínicas de Porto Alegre, Brazil.

A total of 12 C57BL/6 mice (8–11 weeks old) were housed in plastic boxes (30 × 19 × 13 cm) at the Animal Experimentation Unit of the Hospital de Clínicas de Porto Alegre. The mice were kept on a 12-hour light/dark cycle (lights on from 7 AM to 7 PM) at 22 ± 4°C and given free access to food (Purina-Nutripal, Porto Alegre, RS, Brazil) and water.

The mice were randomly divided into two experimental groups (*n* = 6 per group). The groups consisted of mice exposed to intermittent hypoxia for 35 days (IH group) and mice that underwent a simulation of the IH procedure (SIH group). 

**Figure 1:**
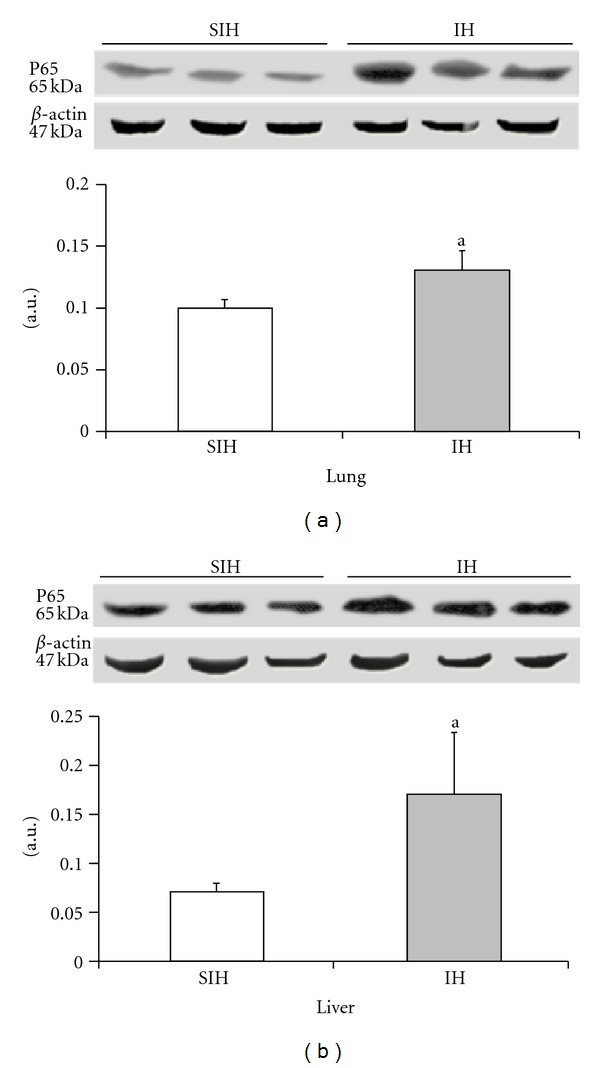
The effect of intermittent hypoxia on the expression of phosphorylated NF-*κ*B in the liver ((a), *P* = 0.0247) and lung ((b), *P* = 0.0033). Results are reported as mean ± standard error, *n* = 6 per group. *P* value according to Student's *t*-test.

The mice were placed in intermittent hypoxia chambers 8 hours a day (9 AM to 5 PM) for 5 weeks (Figure 1). The animals were exposed to a gas mixture consisting of 90% nitrogen and 10% carbon dioxide for 30 seconds. The gas mixture reduces the oxygen fraction in the chambers by 6 ± 1%. In sequence, the gas release is then blocked and fans are triggered to restore ambient air for the remaining 30 seconds. The SIH group was housed in a cage and subjected to the same adjacent fan activity as the IH group but no gas was introduced into the cage [25].

After 35 days, the animals were deeply anesthetized with an intraperitoneal injection of ketamine hydrochloride (100 mg/kg) and xylazine hydrochloride (50 mg/kg) and the liver and lungs were removed. The organs were immediately frozen in liquid nitrogen and kept at −80°C for subsequent analysis. The animals were euthanized by exsanguination under deep anesthesia [26, 27].

The organs were cut and divided for biochemical and protein analyses. For analysis of oxidative stress, 100 mg of tissue was added to 0.9 mL of buffer (140 mM KCl, 20 mM phosphate, pH 7.4) and homogenized with a micropestle in microtubes. After centrifugation at 2150.4 g for 10 minutes in a refrigerated centrifuge (4°C), the supernatant was discarded and the pellet was stored at −80°C for further analysis. For western blotting, a nuclear extraction protocol was used. Briefly, 100 mg of tissue was added to 0.6 mL of lysis buffer (25 mM HEPES, 1% Triton X-100, 2 mM EDTA, 0.1 mL NaCl, 25 mM NaF, 1 mM sodium orthovanadate, and a protease inhibitor cocktail) and homogenized with a micropestle in microtubes. After centrifugation at 15,000 g for 10 minutes at 4°C, the supernatant was discarded and the pellet was stored at −80°C for further analysis.

### 2.1. Oxidative Stress

#### 2.1.1. Proteins

The protein concentration in the homogenate was measured spectrophotometrically at 595 nm using the Bradford method. The values are expressed in mg/mL [28] and were used in the calculations for the TBARS and antioxidant enzymes.

#### 2.1.2. Assessment of Lipid Peroxidation

The TBARS technique consists of heating the homogenate with thiobarbituric acid to produce a colored product that is subsequently measured at 535 nm using a spectrophotometer. The change in color is due to the presence of malondialdehyde and other substances produced from lipid peroxidation in the biological material.

Briefly, 0.25 mL of 10% trichloroacetic acid (TCA), 0.10 mL of homogenate, 0.067 mL of 0.67% thiobarbituric acid (TBA), and 0.033 mL of distilled water were added to a tube, stirred, and then heated at 100°C. After the tubes cooled, 0.20 mL of n-butyl alcohol was added to extract the pigment. The tubes were then stirred and centrifuged for 10 minutes at 1110 g. A 0.20 mL aliquot of the supernatant was added to a 96-well plate. The absorbance of the samples was quantified on a spectrophotometer at 535 nm. The TBARS concentration was expressed in nmol per mg protein [29].

#### 2.1.3. Determination of Superoxide Dismutase (SOD)

The technique used to measure SOD was based on the level of inhibition caused by the reaction of the enzyme with O^−2^. We used adrenaline in an alkaline medium to produce adrenochrome and O^−2^ [30].

In a 96-well plate, we measured SOD activity in the reaction medium (50 mM glycine-NaOH, pH 10) and three samples containing different concentrations of homogenate. After addition of 10.5 *μ*L epinephrine (60 mM, pH 2.0), the reaction was monitored for 2 min at 480 nm. The enzymatic activity was expressed in units SOD/mg protein.

#### 2.1.4. Determination of Catalase (CAT)

Catalase enhances the decomposition of hydrogen peroxide into water and oxygen. The rate of decomposition of hydrogen peroxide is directly proportional to enzyme activity and follows pseudo-first-order kinetics with respect to hydrogen peroxide. 

The decrease in absorption at 240 nm was determined after adding 7 *μ*L of 300 mM H_2_O_2_ to the reaction medium (50 mM phosphate regulator). The catalase concentration was expressed as pmol/mg protein [31].

### 2.2. Western Blots

A total of 50 mg of protein was added to a buffer (60% glycerol, 2 M Tris, SDS, and 10% Pyrroline 0.5%) and incubated for four minutes at 100°C. After electrophoresis was performed [32] on a 9–12% polyacrylamide gel, the protein was transferred to a polyvinylidene difluoride (PVDF) membrane [33]. The membrane was washed with PBS contained 0.5% Tween 20 and then incubated in a blocking solution (5% skim milk powder and 0.5% Tween 20 in cold PBS) for 30 minutes. After washing, the membrane was incubated overnight at 4°C with the primary antibody. Next, the membrane was washed and incubated in the secondary antibody (HRP) for two hours at room temperature. After another wash, the protein was visualized using chemiluminescent detection (Chemiluminescent HRP Substrate), film, and a transiluminator (L-Pix Chemi molecular imaging—Loccus Biotechnology). *β*-actin was used as a loading control. The results were quantified using LabImage 1D (Loccus Biotechnology) and are expressed as arbitrary units.

### 2.3. Statistical Analysis

For analyzing the result, the Student's *t*-test was performed using SPSS version 18.0 (Statistical Package for Social Science). The results are represented as the mean ± standard error of the mean. The statistical significance level was set as *P* < 0.05. 

## 3. Results

Lipid peroxidation, a marker of oxidative damage, was significantly increased in the lung (14%) and liver (29%) of the IH group when compared with the SIH group (Table 1).

The activity of endogenous SOD was significantly lower in lung tissue (56%) and higher in liver tissue (87%) from IH animals when compared with the control group (Table 1). The activity of CAT was significantly higher in both organs (32% in the lung and 184% in the liver) from the IH group when compared with the SIH group (Table 1).

The activated (phosphorylated) p65 subunit of NF-*κ*B was increased by 30% in the lung and 39% in the liver of IH mice when compared with SIH mice.

**Figure 2:**
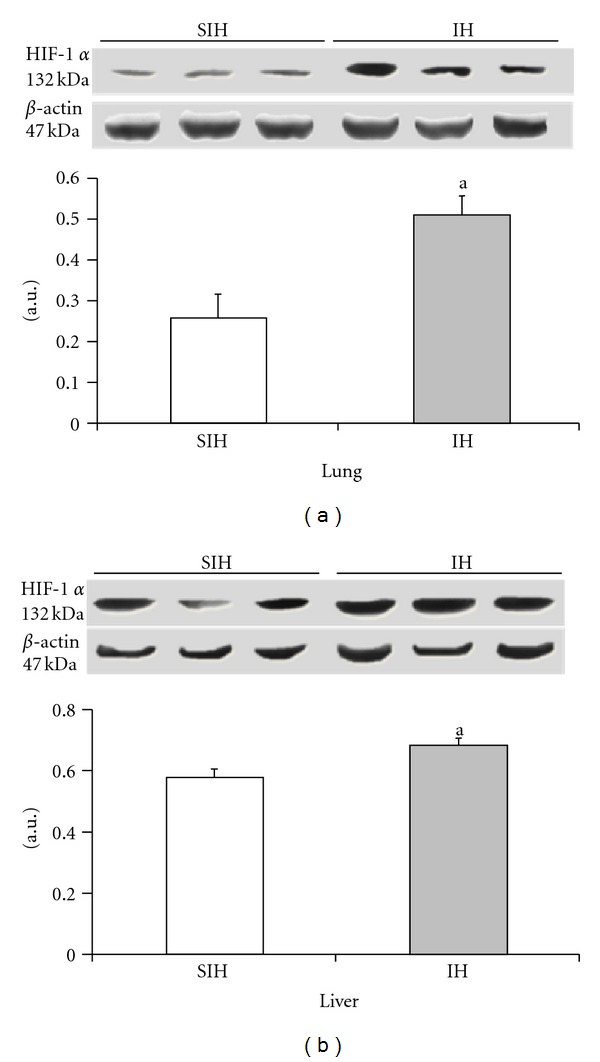
The effect of intermittent hypoxia on the expression of HIF-1*α* in the liver ((a), *P* = 0.0227) and lung ((b), *P* = 0.0086). Results are reported as mean ± standard error, *n* = 6 per group. *P* value according to Student's *t*-test.

**Figure 3:**
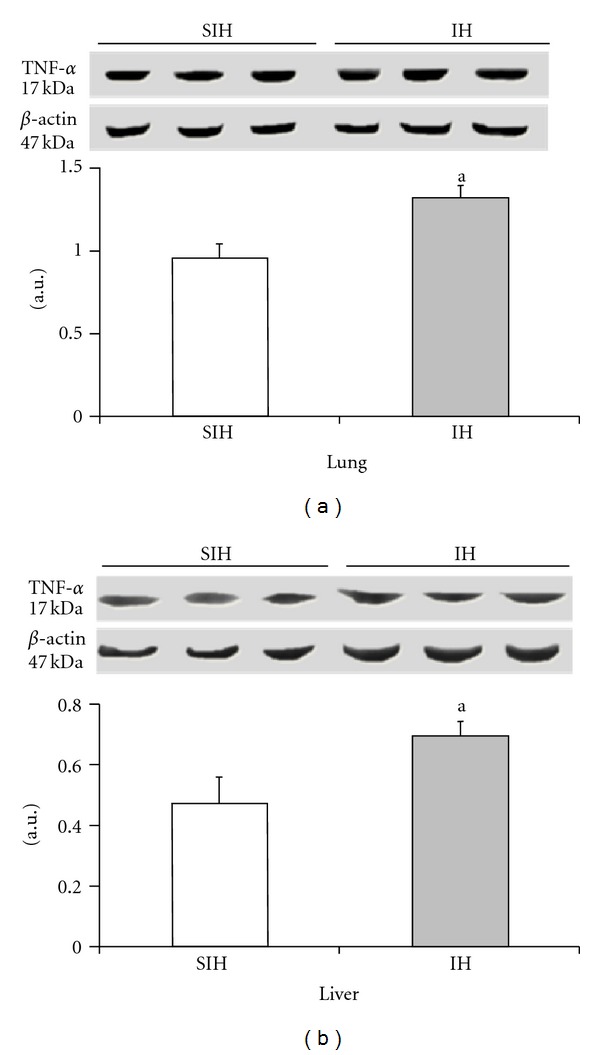
The effect of intermittent hypoxia on the expression of TNF-*α* in the liver ((a), *P* = 0.0382) and lung ((b), *P* = 0.0171). Results are reported as mean ± standard error, *n* = 6 per group. *P* value according to Student's *t*-test.

The expression of HIF-1*α* and TNF-*α* was significantly increased in the IH group when compared with the SIH group (Figures 2 and 3). In the lung tissue, HIF-1*α* increased by 96% and TNF-*α* increased by 38%. In the hepatic tissue, HIF-1*α* was increased by 19% and TNF-*α* was increased by 48%.

**Figure 4:**
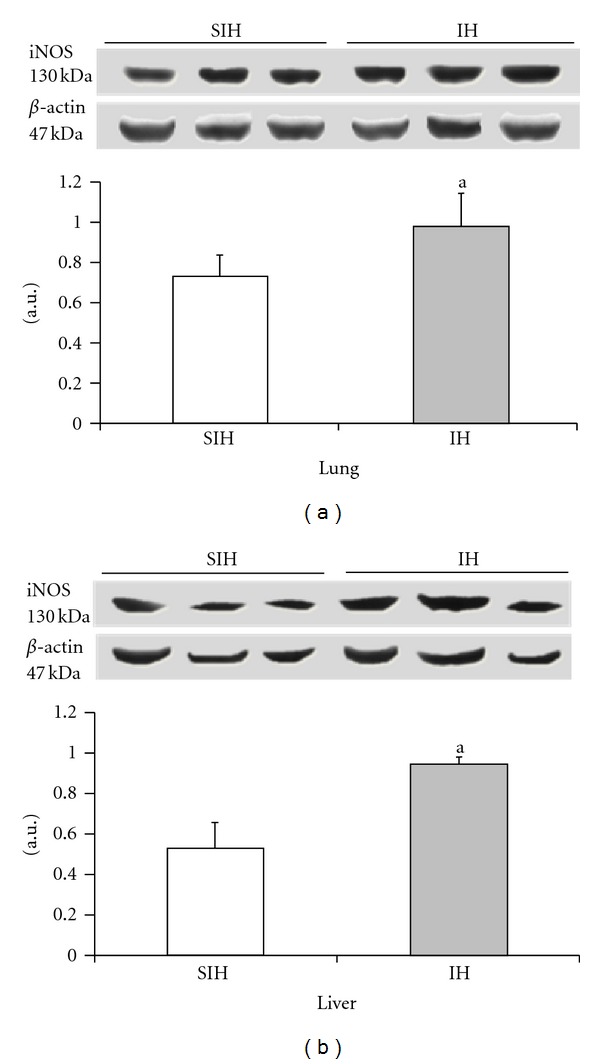
The effect of intermittent hypoxia on the expression of iNOS in the liver ((a), *P* = 0.0091) and lung ((b), *P* = 0.0107). Results are reported as mean ± standard error, *n* = 6 per group. *P* value according to Student's *t*-test.

**Figure 5:**
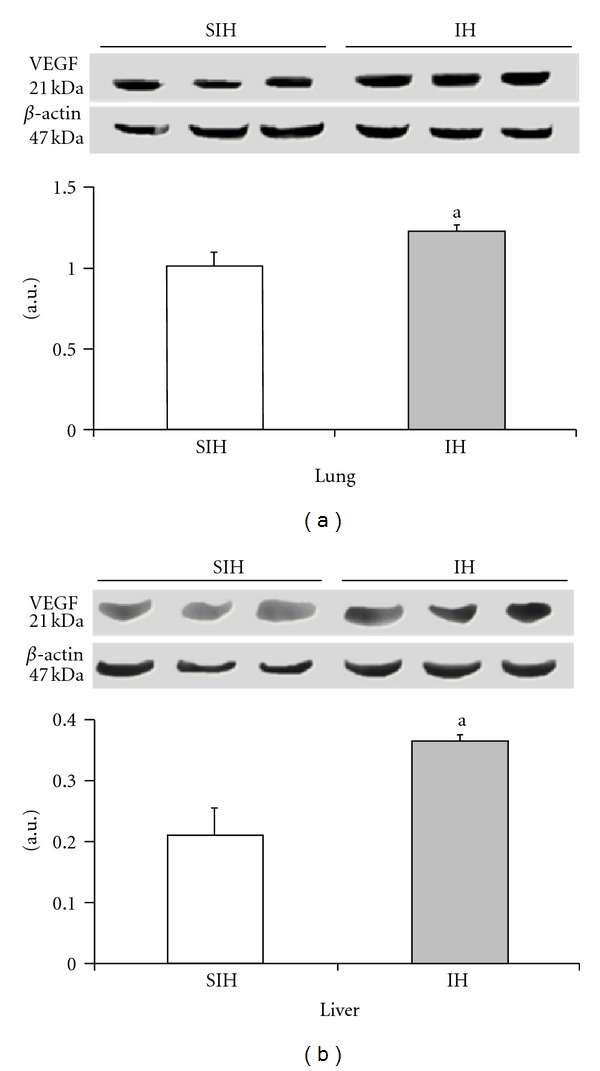
The effect of intermittent hypoxia on the expression of VEGF in the liver ((a), *P* = 0.0062) and lung ((b), *P* = 0.0184). Results are reported as mean ± standard error, *n* = 6 per group. *P* value according to Student's *t*-test.

**Figure 6:**
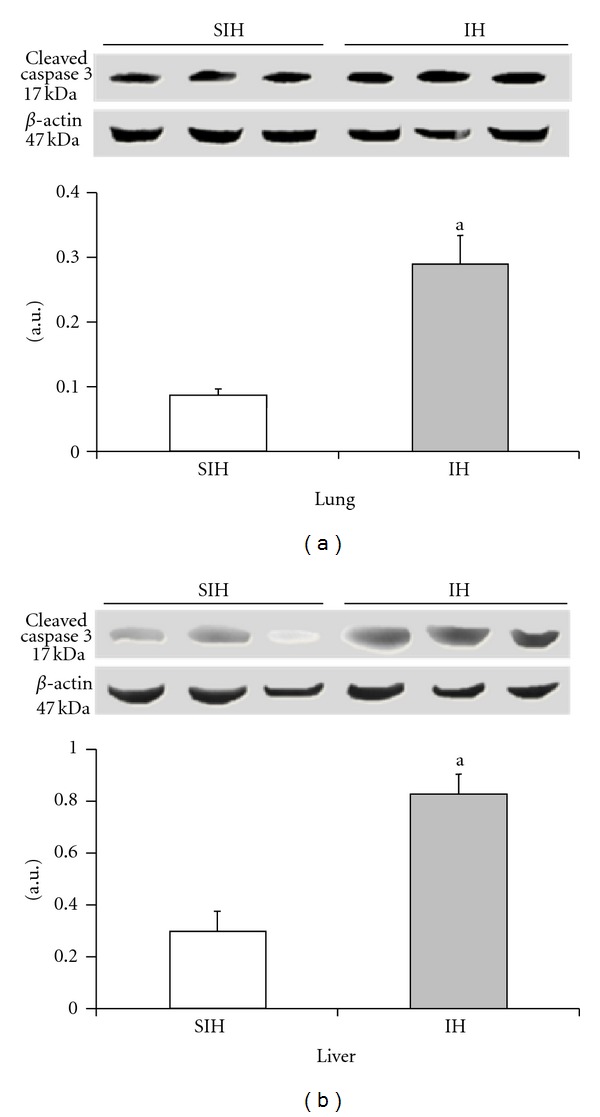
The effect of intermittent hypoxia on the expression of cleaved caspase 3 in the liver ((a), *P* = 0.0022) and lung ((b), *P* = 0.0003). Results are reported as mean ± standard error, *n* = 6 per group. *P* value according to Student's *t*-test.

The expression of iNOS and VEGF was significantly higher in the IH group when compared with controls (Figures 4 and 5). There was a 35% increase in iNOS and a 22% increase in VEGF in the lung tissue. The liver showed a 79% increase in iNOS levels and a 71% increase in VEGF. Cleaved caspase 3 was increased by 237% in the lung and 182% in the liver of IH animals when compared to the SIH group (Figure 6).

## 4. Discussion

Animal models that use intermittent hypoxia can help elucidate the mechanism of damage to various systems caused by sleep apnea. Independent of body mass index, the respiratory disturbance index is directly related to the degree of liver damage and is recognized as a risk factor for nonalcoholic fatty liver disease (NAFLD) [10, 34]. It has been proposed that the development of NASH is produced in two phases consisting first of the accumulation of triglyceride, which is attributed to insulin resistance and obesity, and then the presence of inflammation and fibrosis [35], which is correlated with oxidative stress and hepatic lipid peroxidation [36, 37].

Our research group has described [38] oxidative damage to membrane lipids measured by TBARS and changes in endogenous antioxidant enzymes in the liver tissue that indicate the role of oxidative stress in our model system. These data are in agreement with the results described in other model systems [38–41]. Oxidative stress occurs through xanthine oxidase by producing the superoxide anion radical (O_2_^−∙^) and hydrogen peroxide [42, 43]; it is suggested that the O_2_^−∙^ and H_2_O_2_, formed by the activity of xanthine oxidase, act independently on the activity of SOD and CAT [44]. Nitrosative stress includes the formation of nitric oxide (NO) that binds O_2_^−∙^ to form the radical peroxynitrite [38, 42, 45, 46].

HIF-1*α* regulates the concentration of oxygen, and it can be the initiator of inflammation in intermittent hypoxia [47, 48] or stimulated by oxidative stress [49]. This protein is correlated with chronic alcohol use and the presence of NAFLD [50]. HIF-1*α* also stimulates macrophages, increases the production of VEGF and iNOS [51, 52], reduces apoptosis [24, 53], and stimulates cell proliferation [54].

It is suggested that inflammatory activity is dependent on NF-*κ*B [55], indicating that NF-*κ*B can regulate HIF-1*α* transcription [56]. Although indirect, inhibition of IKK experimentally prevents the activation of NF-*κ*B and was found to prevent the development of steatosis and NASH [57]. In the present study, we observed an increase in the expression of HIF-1*α* in the liver and the lung of mice exposed to hypoxia.

The stimulation of TNF-*α* leads to phosphorylation of I*κ*B, which results in activation of NF-*κ*B. Activation of NF-*κ*B causes it to translocate to the nucleus and promote the transcription of numerous proinflammatory genes [58]. Here, we showed that TNF-*α* and NF-*κ*B were increased in animals exposed to intermittent hypoxia.

VEGF is essential for the initiation of angiogenesis, and it has a strong effect on vascular elements in response to hypoxia [59, 60]. In this study, we found increased expression of VEGF in both organs when mice were subjected to intermittent hypoxia.

In our previous work, we found that there is an increase in nitric oxide metabolites (NO) after exposure to intermittent hypoxia [38]. In the present study, we evaluated an enzyme responsible for NO production, iNOS, and found that the levels of this enzyme were increased in the lung and liver of animals exposed to hypoxia.

Apoptosis in all cells is regulated by caspases. After cleavage, caspases become active and initiate pathways that lead to apoptosis [61]. We found that cleaved caspase 3 expression is increased in the liver and lung of the IH group, demonstrating that there was activation of this apoptotic cascade.

Thus, the data suggest that intermittent hypoxia leads to liver and lung damage that can result from a cascade of signals initiated by oxidative stress, inflammation, and apoptosis.

## 5. Conclusion

In mice, the cyclic oxygen deprivation observed in sleep apnea induces oxidative stress and activation of HIF-1*α*, which stimulates a cascade of inflammatory signaling, nitric oxide generation, angiogenesis, and apoptosis in the lung and liver.

## Figures and Tables

**Table 1:** The effect of intermittent hypoxia on hepatic lipid peroxidation as shown by the TBARS assay and liver antioxidant enzyme activity.

		SIH	IH	*P* value
Liver	TBARS_(nmol/mg prot)_	2.90 ± 0.23	3.76 ± 0.15	0.0389
	SOD_(USOD/mg prot)_	3.13 ± 0.53	5.86 ± 0.70	0.0118
	CAT_(nmol/mg prot)_	0.82 ± 0.17	2.33 ± 0.09	0.0015

Lung	TBARS_(nmol/mg prot)_	4.57 ± 0.10	5.22 ± 0.10	0.0116
	SOD_(USOD/mg prot)_	7.27 ± 0.99	4.64 ± 0.22	0.0272
	CAT_(nmol/mg prot)_	2.62 ± 0.18	3.48 ± 0.13	0.0042

Results are reported as mean ± standard error, *n* = 6 per group. *P* value according to Student's *t*-test.

SIH: sham intermittent hypoxia group; IH: intermittent hypoxia.

SOD: superoxide dismutase; CAT: catalase.
